# Cell Proliferation, Movement and Differentiation during Maintenance of the Adult Mouse Adrenal Cortex

**DOI:** 10.1371/journal.pone.0081865

**Published:** 2013-12-04

**Authors:** Su-Ping Chang, Hamish D. Morrison, Frida Nilsson, Christopher J. Kenyon, John D. West, Steven D. Morley

**Affiliations:** 1 Centre for Integrative Physiology, University of Edinburgh, Edinburgh, United Kingdom; 2 Division of Health Sciences, University of Edinburgh, Edinburgh, United Kingdom; 3 Centre for Cardiovascular Science, University of Edinburgh, Edinburgh, United Kingdom; University of Warwick – Medical School, United Kingdom

## Abstract

Appropriate maintenance and regeneration of adult endocrine organs is important in both normal physiology and disease. We investigated cell proliferation, movement and differentiation in the adult mouse adrenal cortex, using different 5-bromo-2'-deoxyuridine (BrdU) labelling regimens and immunostaining for phenotypic steroidogenic cell markers. Pulse-labelling showed that cell division was largely confined to the outer cortex, with most cells moving inwards towards the medulla at around 13-20 µm per day, though a distinct labelled cell population remained in the outer 10% of the cortex. Pulse-chase-labelling coupled with phenotypic immunostaining showed that, unlike cells in the inner cortex, most BrdU-positive outer cortical cells did not express steroidogenic markers, while co-staining for BrdU and Ki67 revealed that some outer cortical BrdU-positive cells were induced to proliferate following acute adrenocorticotropic hormone (ACTH) treatment. Extended pulse-chase-labelling identified cells in the outer cortex which retained BrdU label for up to 18-23 weeks. Together, these observations are consistent with the location of both slow-cycling stem/progenitor and transiently amplifying cell populations in the outer cortex. Understanding the relationships between these distinct adrenocortical cell populations will be crucial to clarify mechanisms underpinning adrenocortical maintenance and long-term adaptation to pathophysiological states.

## Introduction

The adult adrenal cortex consists of three principal concentric morphological zones, surrounding a central medulla, distinguished by their cellular organisation and steroid hormone products (reviewed in 1). The outer zona glomerulosa (ZG) located just beneath the surrounding mesenchymal capsule contains ovoid cells, arranged into arch-like structures surrounding capillary glomeruli, that synthesise the mineralocorticoid aldosterone. The intermediate zona fasciculata (ZF) is made up of cuboid glucocorticoid-synthesising cells organised in columnar bundles (or fascicles) separated by radial open-pore capillary sinusoids, while cells of the inner zona reticularis (ZR) are embedded in a condensed ‘reticulum’ of interconnecting blood vessels and connective tissue. In most mammals the ZR is defined morphologically, but in humans and some primates it serves the specialised function of making C19 adrenal androgens. In rats and some other species, an additional morphologically-distinct zone, the zona intermedia (ZI), has been described at the boundary between the ZG and ZF ([2] and references therein). In the rat, this has subsequently been termed the ‘undifferentiated zone’ (ZU) because, although cells in this region express some steroidogenic enzymes (e.g. steroid 21-hydroxylase; 21-OH; approved symbol Cyp21a1), they do not express either the ZG-specific aldosterone synthase (AS; approved symbol Cyp11b2) or the ZF-specific 11β-hydroxylase (11β-OH; approved symbol Cyp11b1) [2]. Others have argued, however, that these ZI/ZU cells are part of the ZG, which thus comprises a mixture of both terminally differentiated steroidogenic cells and cells with a less differentiated, more plastic phenotype [3].

Steroidogenic cells of the different adrenocortical zones are thought to originate from one or more self-renewing populations of undifferentiated somatic stem cell progenitors, located somewhere in the outer region of the gland or within the capsule [1,4]. Although cells can divide in all three cortical zones, experimental evidence from rats suggests that under normal physiological conditions most cell proliferation occurs in the outer cortex, after which cells move inwards and are eventually eliminated by apoptosis close to the medulla boundary [5–10].

Radial mosaic patterns in adrenal cortices of chimeric and transgenic mosaic rats and mice [11–16] and radial labelled clones in mice expressing transgenic lineage markers [17] suggest a clonally-related origin for cells of all three adrenocortical zones. It remains possible, however, that different zones could be maintained by separate, radially-aligned stem cell populations that share a common developmental origin [18]. Also, experimental manipulations leading to zone-specific hypertrophy and hyperplasia [2,19,20] and steroidogenic enzyme expression [2,21,22] show that that adaptive responses of the mature adrenocortical zones must be autonomous to allow independent regulation of mineralocorticoid and glucocorticoid steroid hormone production.

There is now considerable evidence that resident populations of relatively undifferentiated adult (somatic) stem cells play essential roles in maintaining many highly regenerative tissues (reviewed in 23,24). The key features of adult stem cells are that they are long-lived, relatively undifferentiated and usually divide asymmetrically, both to self-renew and produce more differentiated cell types. They are also typically slow-cycling and can enter periods of quiescence so that, while stem cells have unlimited proliferative potential, they usually divide relatively infrequently unless the host organ is subject to injury or physiological stress. Many stem cells produce an intermediate cell type, termed transient (or transit) amplifying cells (TACs), which have a lower proliferative potential than a stem cell but typically cycle more rapidly and divide a limited number of times, giving rise to progressively more differentiated offspring. TACs thereby generate a clonal cell lineage of post-mitotic, functionally differentiated cells to replace older and damaged parenchymal cells. The nature and location of putative adult stem/progenitor cells maintaining the adult adrenal cortex have yet to be identified definitively (reviewed in 1,4). However, similar to other tissues, it seems probable that the adrenocortical zones are maintained by co-ordinate regulation of cell division, movement and differentiation in response to different physiological stimuli.

Cell division, cell movement and the location of putative somatic stem cells can be monitored using chromatin-labels, such as the thymidine analogue 5-bromo-2'-deoxyuridine (BrdU) or H2B-GFP, which are incorporated into the DNA of cells undergoing division [24]. Thus, actively dividing cells can be identified with a single BrdU injection (‘pulse’-labelling), while subsequent cell movement is monitored by BrdU ‘pulse-chase’-labelling (BrdU pulse-labelling, followed by various ‘chase’ periods in the absence of BrdU). However, longer labelling and chase periods are needed to identify cells that divide and take up label only infrequently and then retain it by cycling slowly or becoming quiescent (long term BrdU ‘label-retaining cells’; LRCs). LRCs can be distinguished from other cells taking up label but continuing to cycle rapidly, as in the latter case the label quickly becomes ‘diluted’ below the level of detection. One drawback is that not all LRCs are stem cells, since cells that take up label during the last division prior to terminal differentiation will also remain labelled. However, in many tissues, labelled stem cells can be distinguished from differentiated LRCs, because they are non-migratory, do not express genes characteristic of more differentiated cell types and can re-enter the cell cycle and proliferate in response to appropriate physiological stimuli. Thus, in principle, stem cell location can be inferred by tracking the movement of differentiated LRCs in relation to undifferentiated LRCs at their point of origin. Indeed, label retention combined with lineage tracing and clonogenicity assays, has proved remarkably successful for identifying adult stem cells in many tissues [24].

Tritiated thymidine and BrdU pulse-chase labelling have been used previously to show that cell proliferation occurs principally in the outer ZI region of the adult rat adrenal cortex, after which the majority of cells move inwards towards the medulla [5–7]. These techniques have yet to be applied systematically to the mouse where, in the absence of a defined ZI, it is not yet clear whether cell movement following division is predominantly centripetal, or if the adult mouse adrenal cortex has long term LRCs that could be candidate stem cells [18]. Therefore, the principal aims of this study were to combine BrdU pulse-chase labelling with immunostaining for phenotypic steroidogenic enzyme markers (i) to identify cell division patterns and the rate and direction of adult adrenocortical cell movement; (ii) to assess different stages of cell differentiation in the adrenocortical zones; (iii) to distinguish slow-cycling and terminally differentiated BrdU-labelled adrenocortical cell populations by assessing whether or not they are able to proliferate following acute trophic stimulus and (iv) to determine if long-term LRCs are present in the adult mouse adrenal cortex.

## Materials and Methods

### Ethics Statement

All the animal work in this study was approved by the University of Edinburgh Ethical Review Committee (applications PL64-02 and PL21-06) and performed in accordance with institutional guidelines and UK Home Office regulations under project license numbers PPL 60/2887 and PPL 60/3635. All surgery was performed under general anaesthesia and all efforts were made to minimise suffering.

### Mice

Wild-type (C57BL/6 x CBA/Ca)F1 hybrid mice (hereafter abbreviated to F1) were bred and maintained in the Biological Research Facility, University of Edinburgh and killed by cervical dislocation following inhalation of gaseous anaesthetic. 

### BrdU labelling and ACTH treatment

For studies of adrenocortical cell movement, 12-week-old F1 mice were given single i.p. BrdU injections (10mg BrdU/ml saline; 0.2ml/mouse) and then killed four hours later or left for various chase periods before tissue collection. For acute adrenocorticotropic hormone (ACTH) treatment, female F1 mice were pulse-chase-labelled by 1-week infusion of BrdU (50mg BrdU/ml saline; 0.1ml/mouse; Alzet osmotic mini-pump model 1007D) followed by a 6-week chase. Mice (n = 6) were then injected with ACTH (Synacthen, Alliance Pharmaceuticals, UK; 12.5 IU/ kg) or vehicle (0.9% saline) and killed 4 hours later, when the proliferative response to ACTH should be maximal [25]. For identification of LRCs, 12 week old F1 mice were infused with BrdU for 1 week (50mg BrdU/ml saline; 0.1ml/mouse; Alzet osmotic mini-pump model 1007D) or 2 weeks (50mg BrdU/ml saline; 0.2ml/mouse; Alzet osmotic mini-pump model 2002) and killed immediately or left for various chase periods.

### Tissue processing and immunostaining

Adrenal glands were fixed in either 4% paraformaldehyde (4 h) in PBS at 4°C for BrdU immunostaining, or 4% neutral buffered formalin (24 h) for dual-immunostaining and immunofluorescence. Fixed adrenals were paraffin wax-embedded, cut and longitudinal 7-10 µm mid-sections mounted on polysine microscope slides.

For BrdU immunostaining, sections were dewaxed in xylene, washed in ethanol and immersed in 3% hydrogen peroxide in methanol for 30 min at RT to block endogenous peroxidase activity. Sections were then rehydrated in 70% ethanol, washed in PBS, and subjected to heat-induced epitope retrieval (HIER). After blocking in 20% normal rabbit serum in TBS for 30 min, sections were immunostained using a mouse monoclonal anti BrdU antibody (Becton Dickinson, 347580; 1:200), followed by a biotinylated rabbit anti-mouse second antibody (Dako, E0354; 1:200) and then avidin-biotin horseradish peroxidase (HRP) (Dako, K0355; 1:1000) using the 3,3′-Diaminobenzidine (DAB) isopac system as substrate (Sigma, D9015). Sections were finally lightly counterstained with haematoxylin.

BrdU/Ki67 dual-immunostaining was carried out after dewaxing, blocking and HIER as described above, using a sheep polyclonal anti-BrdU antibody (Fitzgerald Industries, 20-BS17; 1:7000), followed by a biotinylated rabbit anti-sheep second antibody (Vector Labs, BA-6000; 1:500) and streptavidin-alkaline phosphatase (Dako, D0396; 1:1000) using Fast Blue (Sigma, F3378; 1mg/ml in 0.1M Tris buffer, pH8.0) as substrate. After further HIER, Ki67 was detected using a monoclonal mouse antibody (Novocastra, NCL-Ki67-MM1; 1:250), followed by a biotinylated rabbit anti-mouse secondary antibody (Dako, E 0464; 1:500) and staining with streptavidin-HRP-DAB. For clarity, fast blue-stained sections were mounted without counterstaining.

Dual-immunofluorescent-staining was adapted from [26], using a Vision Biosystems Bond max staining robot. Sections prepared as described above were blocked in 20% normal goat serum in TBS Tween (TBST). AS and CD31 were detected using rabbit polyclonal antibodies for AS (Celso Gomez-Sanchez, Jackson, USA; 1:100) and CD31 (Abcam, ab28364; 1:1000), followed by goat anti-rabbit peroxidase Fab (Abcam, ab97200; 1:500) and then Tyramide Cy3 (Perkin Elmer). 21-OH was detected by incubating sections first in Rodent Block M, followed by a mouse monoclonal 21-OH antibody (Celso Gomez-Sanchez, Jackson, USA; 1:2000) and Mouse on Mouse Polymer (Abcam, ab127055), and Tyramide Cy3 (Perkin Elmer). For dual-BrdU staining, all sections were subjected to HIER using Bond ER1 retrieval buffer (Leica Microsystems), blocked in 20% normal rabbit serum in TBST for 30 mins and immunostained with sheep polyclonal anti-BrdU antibody (Fitzgerald Industries, 20-BS17; 1:10,000), followed by rabbit anti-sheep peroxidase (Source Bioscience; 1:500) and Tyramide Cy5 (Perkin Elmer). Finally, sections were mounted using Permafluor (Thermo Scientific) and examined under a Zeiss LSM510 metaconfocal microscope.

### Quantitative analysis of BrdU-labelled and Ki67-positive cells

Immunostained adrenal sections were examined with a Zeiss Axioskop 2 compound microscope and calibrated digital images captured with a Nikon Coolpix 995 or JVC 3CCD digital camera, using KS 300 or MCID digital imaging software (Imaging Research Inc).

Following single BrdU injection, positions of individual labelled cells were determined on calibrated digital images of BrdU-stained adrenal sections using ImageJ software [27]. Measurement along the shortest straight line between the outer capsule and the cortical-medulla boundary, passing through the position of a labelled cell was used to determine its distance from both boundaries. To allow for variations in cortical thickness, measured distances of labelled cells from the capsule were then converted to a percentage of the total capsule-medulla distance.

Following ACTH treatment, cells with BrdU-positive, Ki67-positive or BrdU/Ki67-dual-labelled or unlabelled nuclei were counted manually in ZG, outer ZF, inner ZF and ZR fields in digital images of BrdU/Ki67-dual-immunostained adrenal sections. Four sections from left adrenals of 6 control and 6 ACTH-treated female F1 mice were analysed by a single observer who was blinded to treatment. Cells displaying a typically elongated endothelial cell nuclear morphology [28] were excluded from the analysis. Mean percentages (±SEM) of total labelled and unlabelled nuclei numbers were calculated for each zone and treatment. Cell size was estimated by dividing field area by the total number of nuclei within the field (150-200 cells).

After 1 or 2-weeks of BrdU infusion, followed by various chase periods, BrdU-labelled cell numbers were counted in calibrated digital images of adrenocortical sections by superimposing a 10x10 grid on the image to span the full thickness of the cortex. The number of BrdU-labelled cells were then counted separately in each of the 10 grid rows (from the outside of the capsule to the cortical-medulla boundary), using ImageJ. To allow for variations in cortical thickness, data collection was restricted to cortical regions which were between 330-400µm thick and where the flanking adrenal capsule was approximately parallel to the cortical-medullary boundary. BrdU cell counts scored cells as labelled or unlabelled and did not asses variations in staining intensity.

### Statistical analysis

Statistical tests (Student’s *t*-test and χ^2^ test with Yates correction) were performed using Microsoft Excel, StatView (SAS Institute Inc.) or GraphPad Prism (GraphPad Software Inc.). Data were analysed for the effects of ACTH treatment on BrdU-positive, Ki67-positive, BrdU/Ki67-dual-labelled and unlabelled nuclei, using a General Linear Model of ANOVA (Minitab software v12), to take account of any variability between sections and adrenal glands.

## Results

### Dynamics of cell proliferation and movement in the adult adrenal cortex

**Figure 1:**
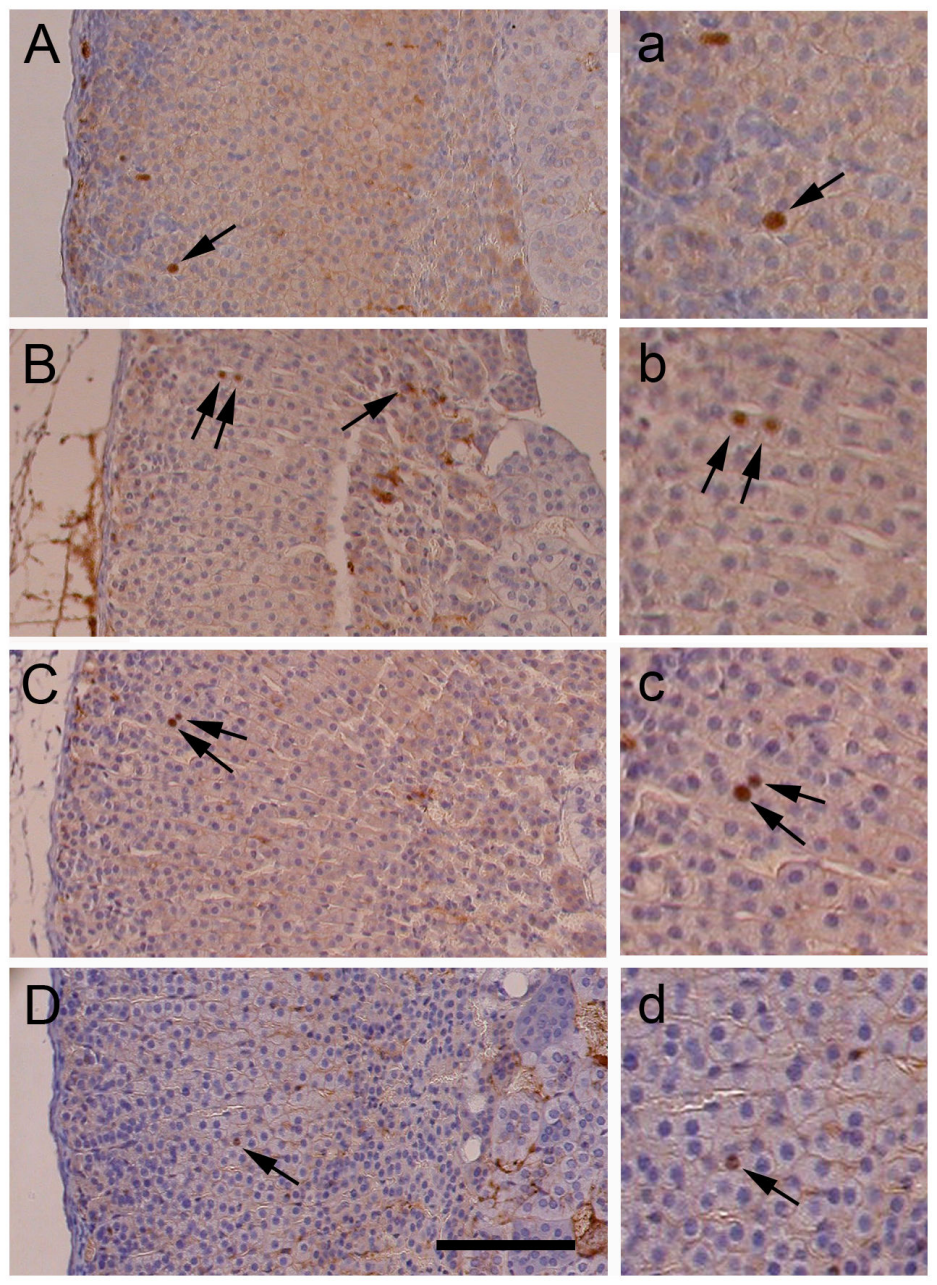
BrdU pulse-labelled cells in adult mouse adrenal glands. (A-D) BrdU immunostaining of adrenal sections from female F1 mice (A) 4 hours; (B) 1 week; (C) 3 weeks & (D) 5 weeks after a single BrdU injection. Typical BrdU-labelled cells (brown nuclei) are indicated by arrows. (a-d) Higher magnification views of BrdU-positive nuclei that are shown in A-D. Scale = 100 µm in A-D; 50 µm in a-d.

**Figure 2:**
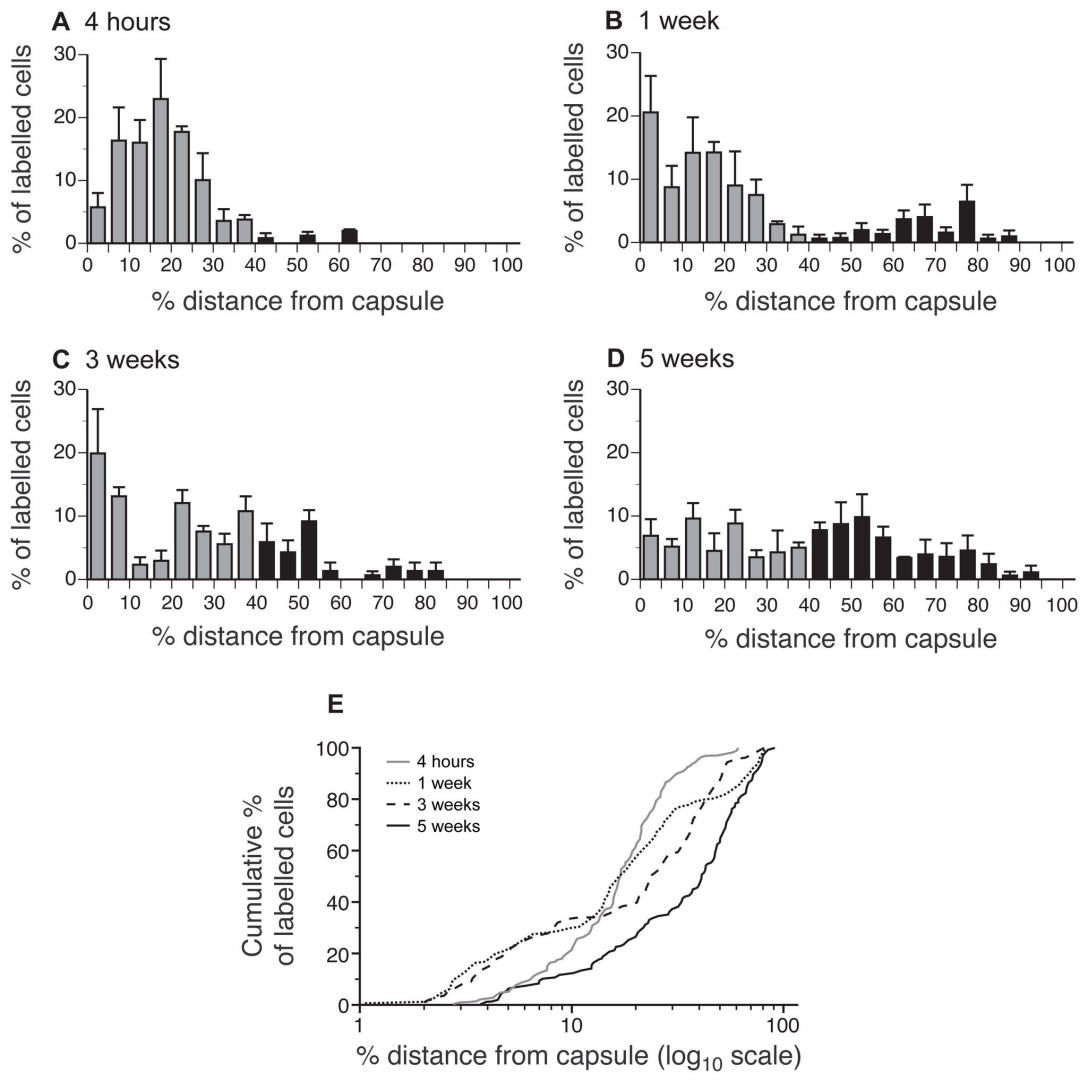
Distribution of single injection BrdU pulse-labelled cells in adult mouse adrenal glands. For quantitative analysis, BrdU-labelled cells were counted in left adrenal mid-sections from 3 mice per group and their positions measured, as described in the Materials and Methods section. Labelled cell numbers per section (mean±SEM) were respectively A. 51.0±5.51; B. 45.7±5.24; C. 37.0±7.94 and D. 48.0±9.07. Cell positions, expressed as the percentage of the radial depth of the cortex from the capsule, were grouped into 20 locations (X-axis in A-D). Unlabelled cells were not counted, so the frequency of labelled cells in each location is expressed as a percentage of all the labelled cells in the section (Y-axis). Grey bars, <40% distance from the outside of the capsule (high proliferative region); black bars, ≥40% distance (low proliferative region). (E) Semi-log cumulative percentage plot of results shown in A-D to focus on cell distributions in the outer 10% of the cortex.

To define the main proliferative region in the mouse adrenal cortex, locations of cells labelled by single BrdU injection were examined in left adrenals of 12-week-old female F1 mice (Figures 1 and 2). Four hours after BrdU injection, most labelled cells (147/153 = 96%) were in the outer 40% of the cortex (Figures 1A and 2A). This is also seen as a change in slope of the cumulative frequency graph at around 40% of the distance from the capsule (Figure 2E). Therefore, the outer 40% of the cortex was classified as the ‘high proliferative region’ while the remainder (over 40% of the distance from the outside of the capsule to the medulla) was designated the ‘low proliferative region’. Within the outer high proliferative region, the main area of cell proliferation was concentrated at 5-25% of the distance from the outside of the capsule (112/147 cells = 76%).

To assess mouse adrenocortical cell movement, locations and BrdU pulse-labelled cell numbers were compared at 4 h and 1 week after single BrdU injection (Figures 1A and 2A vs. Figures 1B and 2B). This showed that over this period the mean number of BrdU-labelled cells per section did not change, (45.7±5.2 at 1 week vs. 51.0±5.5 at 4 h; *P* = 0.522; *t*-test). However, a significantly higher proportion of labelled cells were located in the inner, low proliferative region (21.2% at 1 week vs. 3.9% at 4 h; χ^2^ = 18.67; *P* < 0.0001), implying that cells had moved from the outer, high proliferative region towards the medulla. The frequency of labelled cells within the outer 10% of the cortex did not change significantly during the first week (29.2% at 1 week vs. 20.9% at 4 h.; χ^2^ = 2.23; *P* > 0.05). Nevertheless, there were significantly more labelled cells within 5% of the outside of the capsule (20.4% at 1 week vs. 5.2% at 4 h.; χ^2^ = 14.01; *P* = 0.0002), suggesting that local outward cell movement occurred within the outer 10% of the cortex. This trend for increased BrdU-positive cell numbers in the outer 5% of the cortex after 1 week is also shown by the cumulative percentage plots (Figure 2E) and was maintained at 3 weeks, but is reduced by 5 weeks. Thus, while most BrdU-labelled cells move inwards from the proliferative region towards the medulla, some cells in the outer 10% of the cortex appeared to move in the opposite direction, towards the capsule.

To estimate the rate of inward adrenocortical cell movement towards the medulla, the distances from the capsule of labelled cells closest to the medulla were compared at 4 hours (216 µm) and 1 week (337 µm) after single BrdU injection, after taking account of differences in cortex thickness by conversion to percentage distances from the capsule (61.5% and 85.1% respectively). This represents a difference of 23.6% of the total distance. The mean distance (±SEM) between the capsule and medulla was determined as 399±2.9 µm for all the adrenals analysed (397±4.5 µm for just those analysed at 4 hours and 1 week after BrdU injection), so a movement of 23.6% of this distance represents a movement of approximately 94 µm in 1 week (13.5 µm per day). One problem with estimating the rate of movement of cell closest to the medulla is that positions of BrdU-labelled cells in the low proliferative region were quite variable (Figure 2A, B). Therefore a second estimate was made closer to the 90^th^ centile of the labelled cells, using the positions of the 5 BrdU-labelled cells located closest to the medulla, after first excluding the 10% of labelled cells nearest to the medulla. The mean (and percentage) of these 90^th^ centile cells from the capsule were 139 µm (30.5%) at 4 hours and 238 µm (65.9%) at 1 week after BrdU injection. The percentage difference of 35.4% represents a distance moved of approximately 141 µm per week (20.1 µm per day). Overall, these two estimates suggest an approximate rate of adrenocortical cell movement of 13-20 µm per day. If cells in different parts of the cortex move at different rates, these will be estimates of the maximum rate of movement, because they are based on the leading groups of cells.

### BrdU pulse-chase labelled cells in the outer cortex lack steroidogenic markers

**Figure 3:**
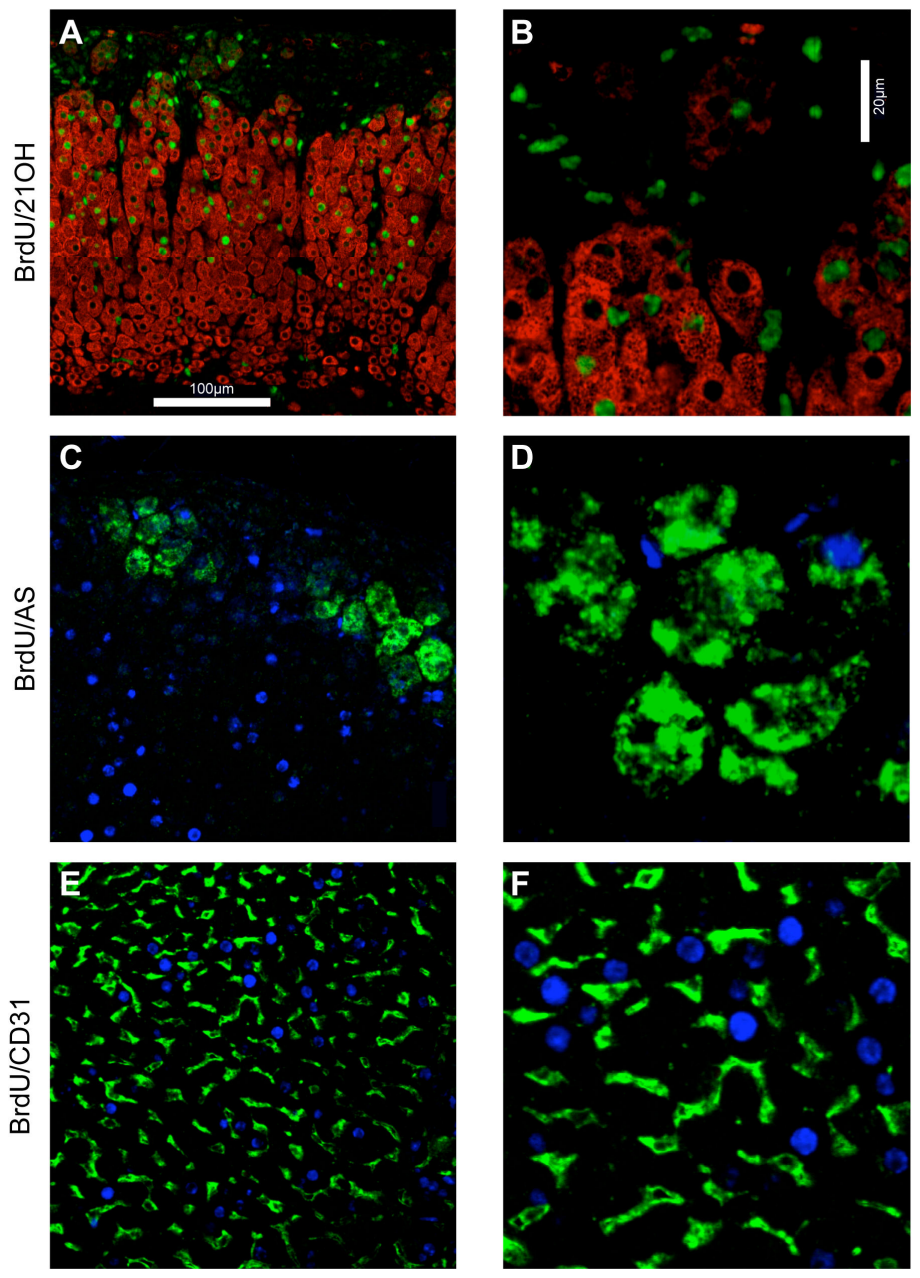
Location of BrdU-pulse-chase-labelled and steroidogenic cells in the adult adrenal cortex. Dual-immunofluorescent staining of adrenal sections from female F1 mice for (A,B) BrdU (green)/21-OH (red); (C,D) BrdU (blue)/AS (green); (E,F) BrdU (blue)/CD31(green), after 1-week BrdU labelling and 6-week chase, as described in the Materials and Methods section. Clusters of weakly staining 21-OH-positive ZG cells near the top in A,B show similar distribution to AS-positive cells in C,D. Scale A,C,E = 100 µm; B,D,F = 20 µm.

Three weeks following single BrdU injection, labelled cells had spread inwards through the cortex from the high proliferative region (Figure 2C) and by 5 weeks were more evenly distributed with some approaching the cortical-medulla boundary (Figure 2D). Based on this and other preliminary data (Figure S1), immunostaining for steroidogenic and vascular endothelial cell markers was combined with a 1 week BrdU pulse followed by 6 weeks chase to analyse the differentiation state of labelled cells in specific adrenocortical zones (Figure 3). BrdU/21-OH dual immunoflourescence showed, as expected, that cells expressing 21-OH, required for synthesis of all three major adrenocorticosteroid classes, were distributed throughout the adrenal cortex (Figure 3A,B), with clusters of weakly stained 21-OH-positive cells in the ZG showing a similar distribution to AS positive cells (Figure 3C). However, most BrdU-labelled cells in the outer cortex did not express 21-OH, while most inner cortical BrdU-labelled cells were positive for 21-OH. AS immunostaining was restricted to arch-like clusters of cells in the ZG though, as might be expected under normal dietary sodium conditions, not all morphologically identifiable ZG cells expressed AS (Figure 3C). The ZG also contained scattered BrdU-labelled cells around the capsule-ZG junction, most of which did not express AS, while most AS-positive cells in the ZG were BrdU-negative (Figure 3C,D). The possibility that much of the BrdU label in the outer cortex might reside in cortical vasculature cells is ruled out by dual-immunostaining for BrdU and CD31, a vascular endothelial cell marker [29]. This showed that large round BrdU-labelled nuclei typical of steroidogenic cells were CD31 negative, while very few CD31-postive cells had BrdU-labelled nuclei (Figure 3E,F). Together, these dual-BrdU/steroidogenic marker-immunostaining results suggest that the majority of BrdU pulse-chase-labelled cells in the outer adrenal cortex lack a differentiated steroidogenic phenotype.

### Response of BrdU pulse-chase-labelled adrenocortical cells to acute ACTH stimulation

**Figure 4:**
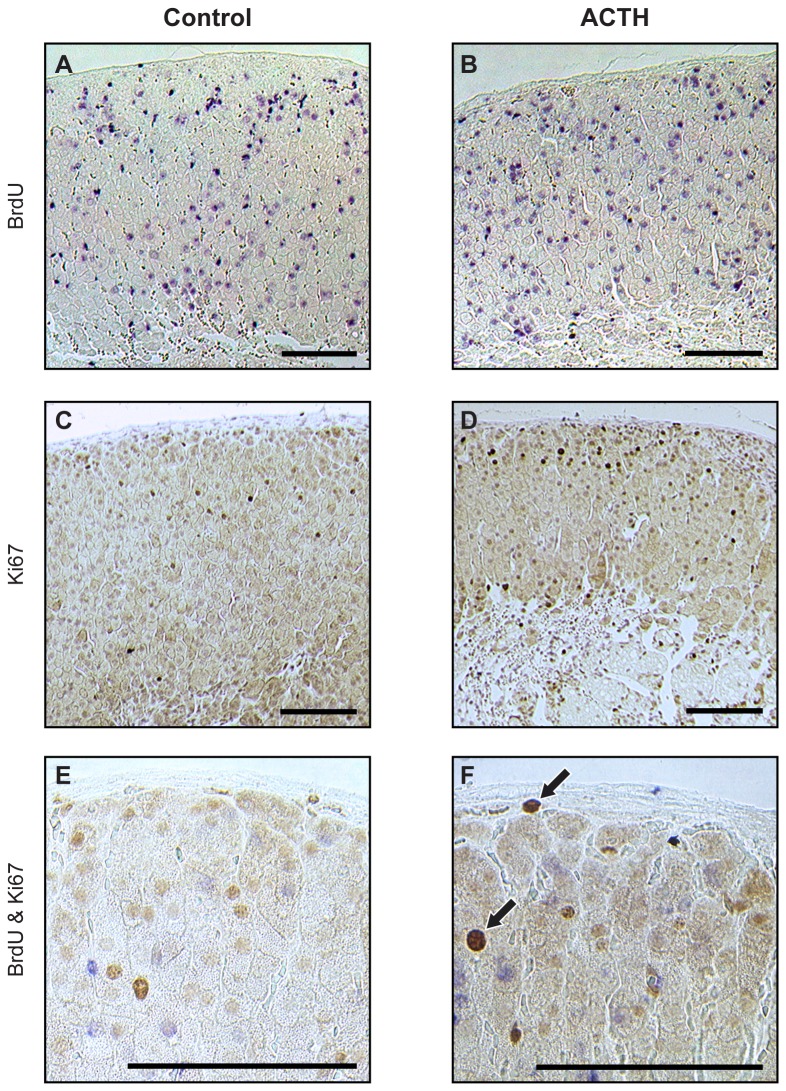
Effect of acute ACTH treatment on BrdU-pulse-chase-labelled cells in the adult mouse adrenal cortex. Adrenal sections from female F1 mice treated acutely with ACTH 6 weeks after 1-week BrdU infusion, immunostained for (A,B) BrdU, (C,D) Ki67 and (E,F) BrdU/Ki67. Mice (n =6/group) were injected with saline vehicle (A,C,E) or ACTH (B,D,F), as described in the Materials and Methods section. Ki67-positive cells are indicated by brown nuclear staining while BrdU-labelled cells are identified by blue nuclear staining. Typical examples of BrdU/Ki67 dual-labelled cells are shown by arrows in F. Sections C and D were lightly counterstained with haematoxylin but sections A,B,E & F were mounted without counterstaining. Scale = 100 µm.

**Figure 5:**
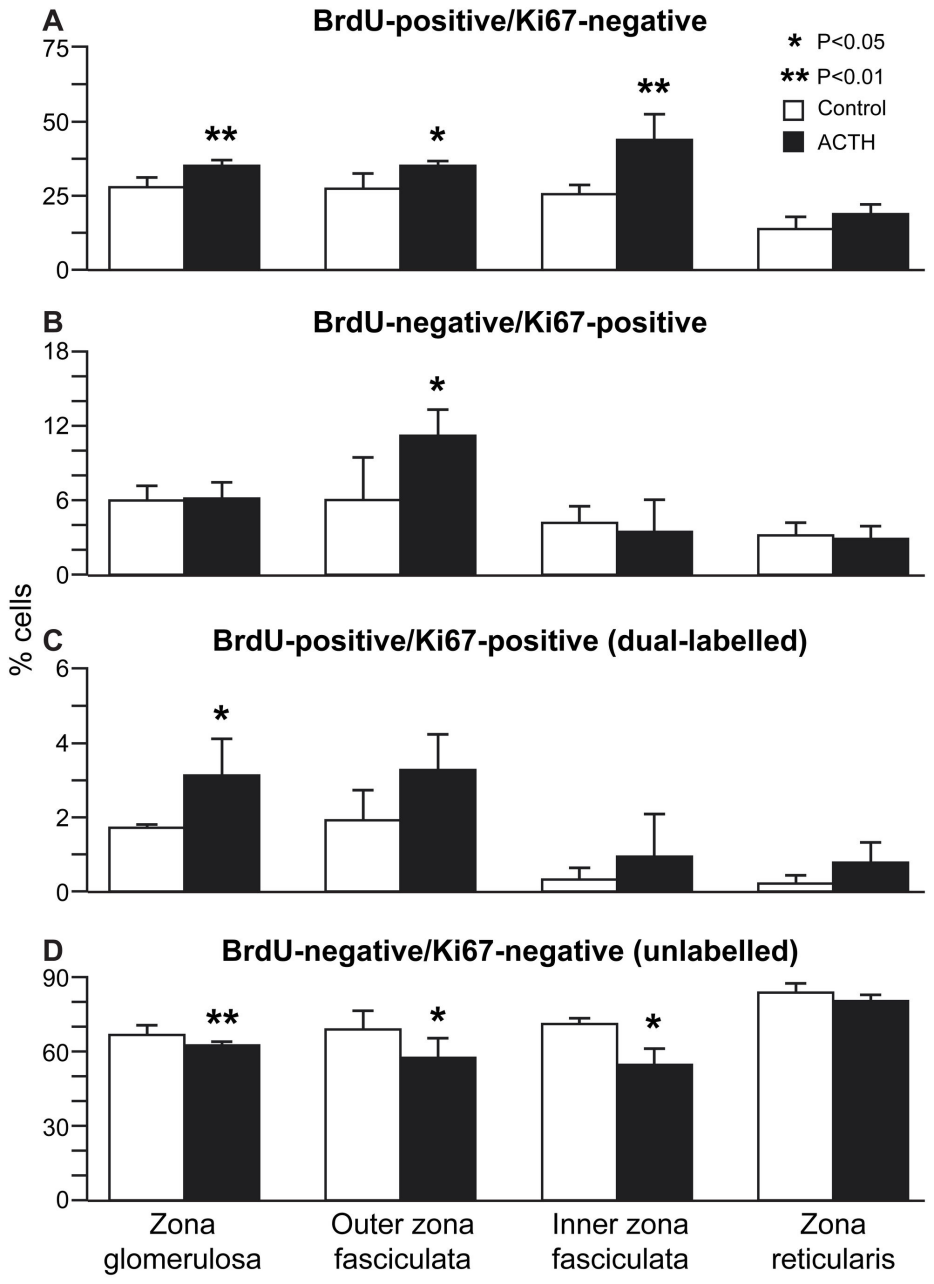
Effect of acute ACTH treatment on BrdU pulse-chase-labelled cell numbers in the adult mouse adrenal cortex. BrdU and Ki67-labelled cell numbers in control and 4 h ACTH treatment groups (n=6) of female F1 mice, treated 6 weeks after 1-week BrdU infusion. Mean percentage (±SEM) of total cell numbers are shown for each zone and treatment that were (A) BrdU-positive/Ki67-negative, (B) BrdU-negative/Ki67-positive, (C) BrdU-positive/Ki67-positive (dual-labelled) and (D) BrdU-negative/Ki67-negative (unlabelled). Cells were counted in ZG, outer ZF, inner ZF and ZR in dual-BrdU/Ki67-immunostained left adrenal sections, as described in the Materials and Methods section.

To distinguish between slow-cycling and terminally differentiated cells in specific adrenocortical zones, BrdU pulse-chase-labelled cells were assessed for their ability to proliferate following acute ACTH stimulation, by dual-staining for BrdU and Ki67. This showed that 4 hours after ACTH treatment, BrdU-labelled cells remained widely distributed throughout the adrenal cortex, but nuclei positive for the Ki67 cell proliferation marker were found mostly in the outer cortex (Figure 4). Quantitative analysis showed that BrdU-labelled cell numbers showed a moderate increase in the ZG and outer and inner ZF following ACTH treatment, while Ki67-positive cells were increased significantly in the outer ZF (Figure 5A-B). Crucially, BrdU/Ki67 dual-labelled cell numbers showed a statistically significant increase in the ZG, with a similar, but non-significant (*P* = 0.14), increase in the outer ZF (Figure 5C). Cell proliferation in response to ACTH was not affected by prior BrdU labelling, since the proportions of Ki67-positive cells did not differ significantly between BrdU-labelled and unlabelled cells in any of the zones before and after ACTH treatment (*P* > 0.1), while acute ACTH-treatment had no significant effect on cell size (average cross-sectional area) in any zone (data not shown).

### Identification and distribution of label-retaining cells in the adult mouse adrenal cortex

Two experiments were undertaken to determine if long term LRCs are present in the adult mouse adrenal cortex, first to optimise the BrdU-pulse-chase period and second to identify LRCs. In a preliminary experiment, 12-week old female F1 mice were infused with BrdU for 1 week, after which the locations of labelled cells in left adrenal glands were examined immediately, or following ‘chase’ periods of 5, 7, 12 and 15 weeks (one mouse per chase period). As expected from the previous single-injection experiment (Figures 1 and 2), most labelled cells were located in the outer cortex immediately following a one-week BrdU infusion (Figures S1 and S2). Following a 5-week chase period, labelled cells had spread to the inner cortex and overall BrdU labelled cell numbers had increased slightly, perhaps indicating that some cells had divided at least once more following incorporation of BrdU. With longer chase periods (7, 12 & 15 weeks; Figures S1 and S2) frequencies of BrdU-labelled cells declined, but even after 15 weeks labelled cells remained relatively abundant and were not restricted to a specific region, implying that longer chase times would be required to identify LRCs in the adrenal cortex.

**Figure 6:**
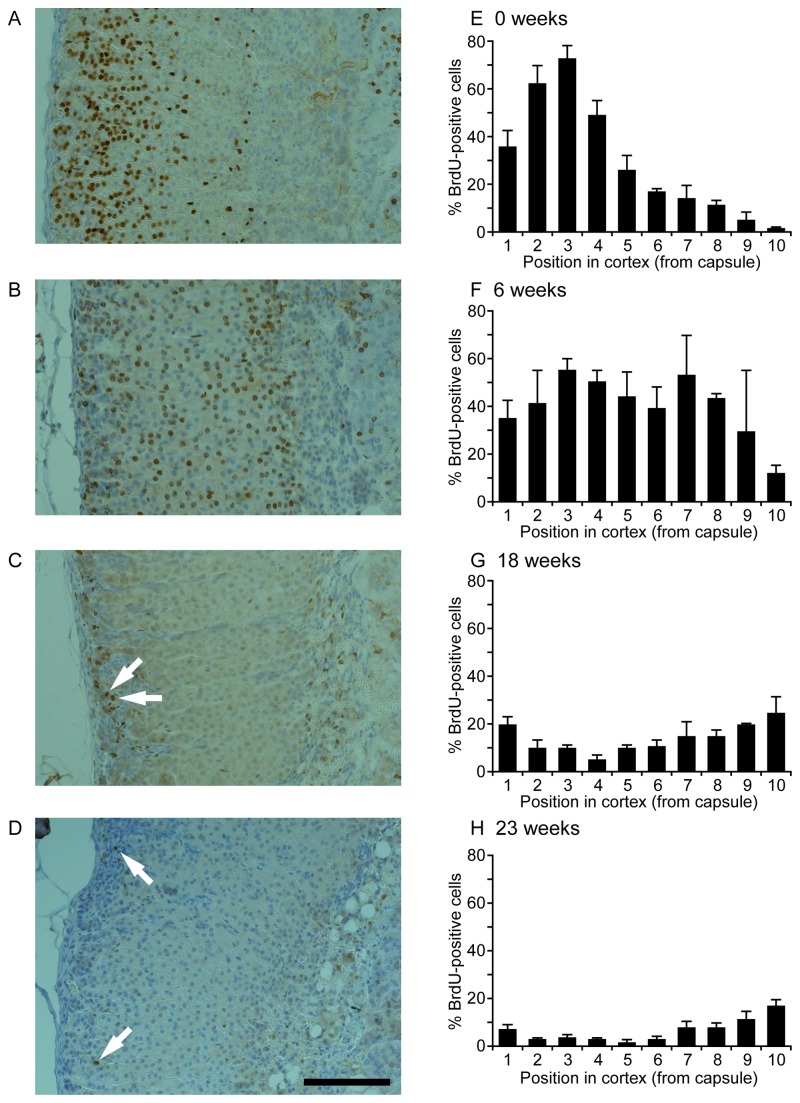
Distribution of BrdU pulse-chase-labelled cells in the adult mouse adrenal cortex following different chase periods. (A-D) BrdU immunostaining of adrenal sections from female F1 mice, (A) 4 hours (n=2), (B) 6 weeks (n=2), (C) 18 weeks (n=3) and (D) 23 weeks (n=5) following 2-week BrdU infusion. Arrows in C,D indicate examples of labelled cells, which at 18 weeks were often clustered near the capsule. Scale = 100 µm. (E-H) Labelled cell distributions are shown as mean percentage (±SEM) of BrdU-positive cells in each of 10 sampling rows across the depth of the cortex (as a percentage of all the cells in the row), in left adrenal sections. Sampling positions 1-10 were rows 1-10 of a 10x10 sampling grid with row 1 including the capsule and row 10 being closest to the medulla, as described in the Materials and Methods section.

Therefore, to address the requirement for longer chase times and to ensure that a greater number of potential LRCs were labelled, in a second experiment 12-week old female F1 mice were infused with BrdU for 2-weeks and the locations of labelled cells in left adrenal glands examined immediately or following different ‘chase’ periods. A 6-week chase period was used to confirm that labelled cell distributions followed those seen in the preliminary experiment (Figures S1 and S2), after which 18 and 23 week chase periods were used to identify LRCs. (BrdU pulse-chase-labelled cells in male left adrenal glands showed similar distributions to females and were not analysed quantitatively.) Again, large numbers of BrdU-labelled cells were detected immediately after the infusion period, being located mainly in the outer cortex (Figure 6A,E). After a 6-week chase period, labelled cells had spread through most of the cortex (Figure 6B,F), consistent with the preliminary experiment, though, subjectively, BrdU staining intensity seemed weaker in some cells. Between 6- and 18-week chase periods the overall frequency of BrdU-labelled cells fell significantly from 37.9% to 14.2% (*P* = 0.002; *t*-test). Following an 18-week chase period most BrdU-labelled cells were located either in the outer cortex, often clustered near the capsule, or in the inner cortex close to the medulla; with fewer labelled cells in the middle of the cortex (Figure 6C,G). A few labelled cells also persisted within the medulla. Following a 23-week chase period, the proportion of BrdU-labelled cells in the cortex fell further to 6.1%, which was significantly less than the 18-week frequency (*P* = 0.007; *t*-test). At this stage, while the majority of remaining BrdU-labelled cells were located in the inner cortex (Figure 6H), there were still a few strongly-staining BrdU-positive cells located mainly close to or in the capsule (Figure 6D). Subjectively, most of the cells retaining a BrdU label in the inner cortex were less strongly-stained than those near the capsule.

## Discussion

In this study, we have attempted to characterise some of the mechanisms involved in adult adrenocortical tissue maintenance by analysing patterns of cell proliferation, movement and differentiation in the adult mouse adrenal cortex.

### Cell proliferation and movement

Single BrdU injection showed that cell proliferation occurred primarily in the outer 40% of the adrenal cortex, with the main proliferative area concentrated at 5% to 25% from the capsule, encompassing the boundary between the ZG and ZF. This is consistent with previous reports in the rat [5–7] and whole-organ labelling in mouse [30]. However, the rate for mouse adrenocortical inward cell movement, of 13-20 µm per day, reported here is greater than that estimated for the rat, which equates to 2.6-5.0 µm per day [6]. This difference may be partly methodological, because we compared locations of cells that were closest to the medulla or at the 90^th^ centile at 4 h and 1 week after pulse-labelling, instead of mean or median positions of labelled cells over longer intervals. Therefore, our estimate will be close to a maximum rate for adrenocortical cell movement, but should minimise errors occurring over longer periods due to increases in labelled cell numbers due to further cell division followed by losses due to label dilution or cell death, and the confounding effects of outward movement in the ZG (discussed below). As far as we are aware, this is the first quantitative estimate of the rate of inward cell movement in the mouse adrenal cortex.

One week after BrdU pulse-labelling most labelled cell movement was towards the medulla but there was also evidence that a minor cell population remained in the outer 10% of the cortex, perhaps moving outwards towards the capsule. Thus, separate TAC populations may replenish the ZG and ZF/ZR. As mentioned previously, distributions of labelled cells over longer periods are more difficult to interpret due to concomitant cell division, movement and cell death. However, persistence of labelled cells in the outer 5% of the cortex at 3 weeks followed by a reduction at 5 weeks could indicate either the presence of specific progenitors that continue to divide or the elimination of terminally differentiated ZG cells by apoptosis or redistribution to the inner cortex following transdifferentiation.

### Adrenocortical cell phenotypes

Under basal conditions six weeks after 1-week BrdU infusion, less than 10% of BrdU-labelled cells in all zones were BrdU/Ki67 dual-labelled, implying that most proliferating cells incorporating BrdU during 1-week pulse then either cycled slowly or underwent terminal differentiation and withdrew from the cell cycle during the six week chase. Most BrdU-labelled cells located in the inner cortex were also positive for 21-OH (expressed in all steroidogenic cells of the adrenal cortex), suggesting that they might be steroidogenic cells which had taken up label prior to terminal differentiation. In contrast, the majority of BrdU-labelled cells in the outer cortex expressed neither 21-OH nor AS (normally expressed exclusively in ZG steroidogenic cells), showing that they were relatively undifferentiated. A further probe of cell phenotype by acute (4 h) ACTH treatment produced a moderate increase in total BrdU-labelled cell numbers in the ZG and throughout the ZF, suggesting division of previously-labelled cells, but total Ki67-positive cell numbers showed a significant increase only in the outer ZF, but not in the ZG or inner ZF. However, BrdU/Ki67 dual-labelled cell numbers were increased significantly in the ZG following ACTH treatment, with a similar trend in the outer ZF. This suggests (i) that the proliferative response to acute ACTH treatment is mainly confined to the previously-identified high proliferative region in the ZG and outer ZF and (ii) that some of BrdU-labelled cells in this region (labelled during proliferation 6 weeks previously, followed by slow-cycling or terminal differentiation) had been induced to proliferate by acute ACTH treatment and become Ki67-positive (current proliferation). This is consistent with the idea that the ZG may contain slow-cycling stem or progenitor cells that are activated in response to physiological stimulus.

As most ZF cells express 21-OH, the majority of Ki67-positive cells in the outer ZF are likely to be partially differentiated TACs, rather than stem or progenitor cells. Also, the increase in Ki67-positive cell numbers following acute ACTH treatment is consistent with an expansion in TAC numbers. ACTH treatment also caused a significant increase in BrdU-labelled cells in the inner ZF, apparently in the absence of increased cell proliferation. Since, most BrdU-labelled cells in the inner ZF also express 21-OH, they are unlikely to be stem/progenitors or TACs but, rather, they may be fully differentiated ZF cells which incorporated BrdU label earlier during terminal differentiation and moved inwards. Meanwhile the ACTH-induced increase in BrdU-labelled cell numbers might be explained by increased cell proliferation in the outer ZF and further inward displacement of labelled terminally differentiated cells.

### Identification of label-retaining cells in the outer adrenal cortex

The observation of two groups of BrdU-labelled cells at 18 and 23 weeks following 2-week BrdU infusion, demonstrates the presence of long term LRCs in the mouse adrenal cortex. LRCs in the outer cortex stained strongly for BrdU but were relatively few in number and located mainly close to or within the capsule. It seems unlikely that these LRCs could be steroidogenic ZG cells which incorporated label during terminal differentiation, because, as noted previously, six weeks after BrdU-pulse-chase labelling most labelled cells in the outer cortex lacked a differentiated steroidogenic phenotype. Therefore, these LRCs may be candidates for slowly cycling adrenocortical stem or progenitor cells. However, additional experiments will be required to determine if they correspond to the subpopulation of BrdU-pulse-chase-labelled cells in the ZG that respond to acute ACTH stimulation by proliferating. The second, less-strongly-staining group of LRCs located in the inner cortex close to the medulla boundary, are probably the last of the inwardly moving cells with their origin in TACs which had divided and incorporated label during the 2-week BrdU pulse, perhaps then undergoing one or two further cell divisions before terminally differentiating and moving inwards towards the medulla during the chase period. It seems unlikely on morphological grounds that these LRCs are located within a persistent X zone and also because female mice were infused with BrdU from 12 to 14 weeks of age when little or no cell division occurs within the X zone [31].

### Stem/progenitor cell maintenance of the adult mouse adrenal cortex

Most adult stem cells characterised to date are non-migratory, lack the differentiated hallmarks of the host tissue and typically cycle slowly, but are able to proliferate in response to physiological stimuli, giving rise to lineage-committed progenitor cells. Experiments presented here using different pulse-chase regimens have identified cell populations in the adult mouse adrenal cortex matching some criteria for putative stem/progenitor cells. Data also support the existence of Ki67-positive, partly differentiated cells in the outer cortex that could be TACs and non-proliferative 21-OH-positive cells in the inner cortex, which are likely to be terminally differentiated steroidogenic cells. Together, this suggests a successively more differentiated cell hierarchy in which subcapsular stem/progenitors and TACs in the outer cortex maintain the differentiated adrenocortical zones.

**Figure 7:**
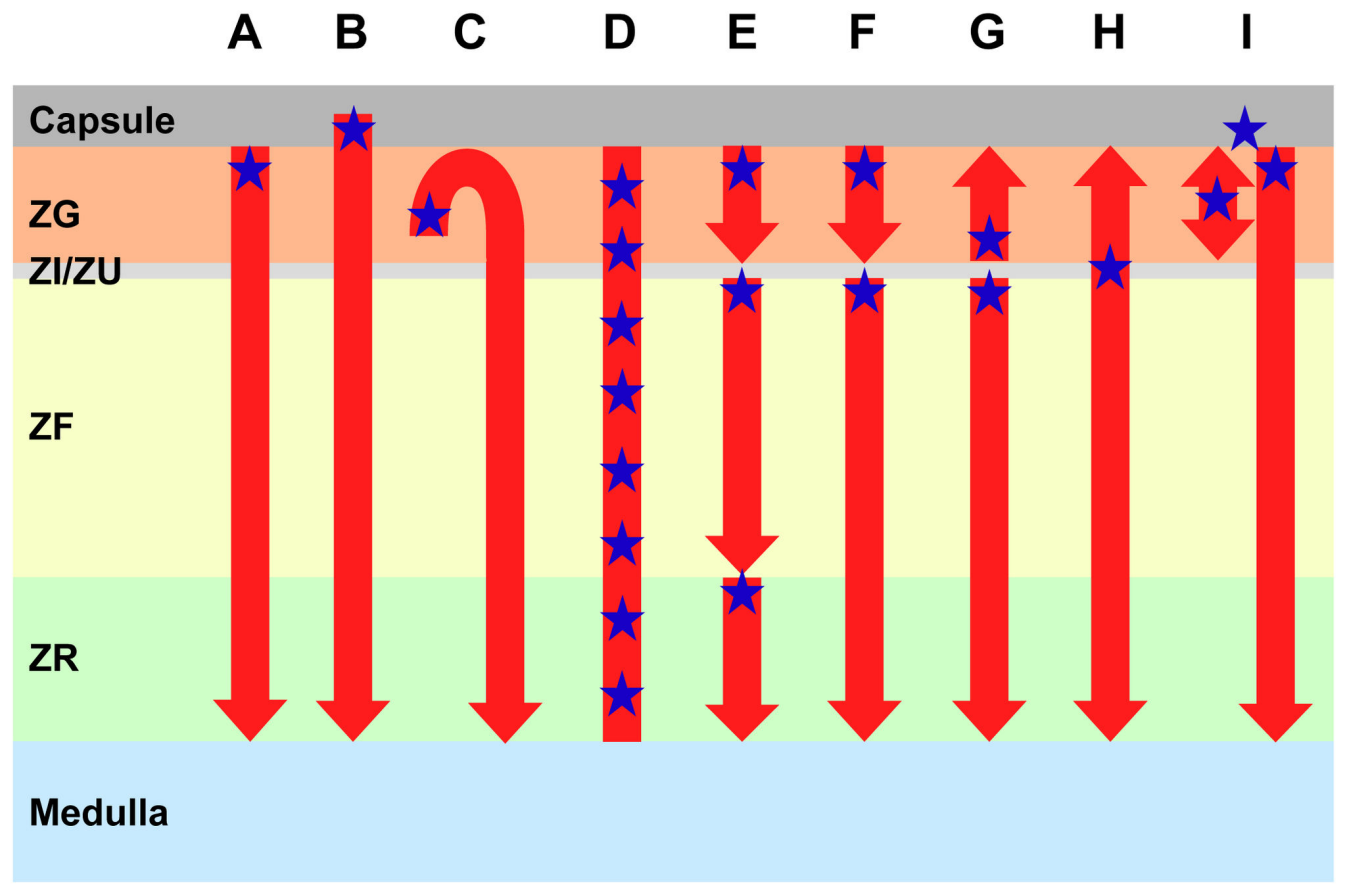
Alternative hypotheses proposed for stem cell maintenance of the adrenal cortex. Each hypothesis is modified to include putative stem cells (blue stars) that produce TACs which divide, the offspring of which move in the direction of the vertical arrows, gradually differentiating and eventually dying near the arrowheads. (A) Classical centripetal migration hypothesis: a single stem cell population in the outer cortex gives rise to new TACs that displace existing cells inwards, transdifferentiating from ZG to ZF to ZR to maintain all three zones (reviewed in 37); (B) Variant centripetal migration hypothesis: similar to (A), except that stem cells are located in the adrenal capsule (reviewed in 1); (C) “Intermediate field” hypothesis: variant of (A) in which stem cells located at the tips of looped strings of cells close to the ZG/ZI boundary, give rise to TACs that move along the strings, first outwards towards the capsule and then arc around to move inwards, following a trajectory shaped like a ‘walking stick’ (reviewed in 37); (D) Zonal hypothesis: each cortical zone is maintained independently by resident stem cells (reviewed in 37); (E) Independent inward migration in three zones: variant of (D) with inward cell movement; (F) Independent inward migration in two zones: variant of (E) where ZF and ZR are maintained by a single stem cell population; (G,H) ZI stem cells: where either two separate stem cell populations or a single bipotential stem cell population in the ZI maintain both the ZG and ZF/ZR [2]; (I) Two separate lineages from outer stem cells: variant hypothesis proposing that stem cells in the outer cortex or capsule produce TACs, which either differentiate into ZG cells or move inwards to become ZF/ZR cells. The ZG and ZF/ZR lineages may be produced either by a single bipotential population or two separate populations of stem/progenitor cells. Unlike (A-C), differentiated steroidogenic ZG cells do not transdifferentiate into ZF/ZR cells.

Our investigations of cell proliferation and movement provide evidence bearing on the different hypotheses proposed for maintenance of the adult adrenal cortex, summarised in Figure 7. Data presented here for mouse and in the literature for rat, fit well with the variant inward migration hypotheses outlined in Figure 7A-C. All of these schemes require transdifferentiation of inwardly moving steroidogenic cells from ZG to ZF/ZR, but only version C is consistent with outward cell movement in the outer cortex. The original zonal hypothesis (Figure 7D) can be discounted because it does not take account of long-range inward cell movement. Zonal variants proposing inward movement from resident stem cell populations within adrenocortical zones (Figure 7E, F) do not accord with pulse-labelling patterns reported here or in whole-organ labelling [30], as these hypotheses require cell movement from several points of origin and predict that LRCs should occur in the ZF/ZR as well as the ZG. Although proliferating cells around the mouse ZG/ZF boundary and the rat ZI/ZU are most likely to be TACs, this region could also contain either 1 or 2 populations of stem/progenitor cells producing separate population of cells that move outwards to maintain the ZG and inwards to maintain the ZF/ZR (Figure 7G,H). However, the presence of BrdU LRCs in the outer ZG but not in the central cortex or at the ZG/ZF boundary, where stem cells would be predicted in hypotheses D and E-H respectively (Figure 7), argues against this, at least for mouse. The present observations can perhaps best be reconciled in the variant cell migration hypothesis shown in Figure 7I in which one or two stem/progenitor cell populations located in the subcapsular ZG and/or the capsule itself, support lineage-committed TACs located around the ZG/ZF boundary, which either differentiate into ZG cells, or move inwards to become ZF/ZR cells. This is consistent with the observation in rat of two distinct proliferative regions surrounding the ZI, which may correspond to two TAC populations responding to different circadian cues [25] and also with elegant lineage tracing experiments favouring two distinct lineages of Shh-positive and Gli1-positive steroidogenic progenitor cells during adrenocortical development [17], possibly supported by a capsular stem cell [1].

The suggestion of two distinct populations of lineage-committed TACs responding to independent physiological stimuli, accords with independent regulation of the adult adrenocortical zones and ZG- and ZF-specific expression of AS and 11β-OH, which allows the gland to respond to different physiological demands. Thus, increased glucocorticoid production regulated by the HPA axis in response to chronic physiological stress is met in the ZF/ZR by increased cell proliferation, hypertrophy and steroidogenic enzyme expression, and reduced apoptosis. Similarly, ZG cells numbers expressing AS are low under sodium-replete conditions but increase following salt restriction, when renin-angiotensin system-mediated aldosterone production is required to promote sodium retention in the kidney. This divergent physiological regulation indicates that several key switches will be required to direct adrenocortical steroidogenic progenitors to their appropriately differentiated fates.

Evidence for successive steps in steroidogenic cell differentiation is also seen during both adrenocortical development and regeneration. The key steroidogenic transcription factor SF1 is first expressed in the mouse adrenogonadal primordium around E9.5, while 21-OH is detected around E11.5 specifically in the adrenal (but not the gonadal) primordium, and 11β-OH and AS expression are first seen respectively around E12.5 and E16.5, corresponding to the onset of zonation [32–34]. Adrenocortical cell proliferation following surgical enucleation is accompanied by reductions in plasma corticosterone (requiring 21-OH and 11β-OH) and aldosterone (requiring 21-OH and AS) and a transient increase in deoxycorticosterone (requiring 21-OH, but neither 11β-OH nor AS) [35,36]. These observations suggest an initial proliferation of partially differentiated cells expressing 21-OH, before re-expression of 11β-OH and AS following re-establishment of zonation [36]. This is consistent with the idea that partly differentiated TACs around the ZG/ZF boundary express early steroidogenic pathway enzymes including 21-OH, but that terminal differentiation is accompanied by expression of either 11β-OH in the ZF/ZR or AS in the ZG.

## Conclusions

The experiments reported here in the adult mouse adrenal cortex provide evidence for (i) a population of proliferative cells in the outer cortex that moves inwards through the ZF/ZR and a second population in the outer cortex/ZG, representing either slowly dividing progenitors or differentiated ZG cells that are subsequently eliminated by apoptosis or redistribution to the inner cortex following transdifferentiation; (ii) proliferative cells in the outer cortex that could be TACs and non-proliferative 21-OH-positive cells in the inner cortex, which may be terminally differentiated steroidogenic cells; (iii) a subpopulation of BrdU-pulse-chase-labelled cells in the outer cortex that lack steroidogenic markers but divide in response to ACTH; and (iv) LRCs remaining in the outer cortex that retain a BrdU label for up to 18-23 weeks. These observations are consistent with a model in which the adult mouse adrenal cortex is maintained by slow-cycling stem/progenitors giving rise to partially differentiated TACs that proliferate around the ZG/ZF boundary, differentiate and move from the outer cortex to replenish the three adrenocortical zones. If some of the undifferentiated ACTH-responsive BrdU pulse-chase-labelled cells are long term LRCs, they will be candidates for slow-cycling subcapsular stem/progenitor cells.

## Supporting Information

Figure S1 **Preliminary BrdU pulse-chase experiment to optimise the chase period for identifying label-retaining cells.** (A-E) BrdU immunostaining of adrenal sections from female F1 mice, (A) 4 hours, (B) 5 weeks, (C) 7 weeks, (D) 12 weeks and (E) 15 weeks following a 1-week BrdU infusion, showing distributions of BrdU-labelled cells (identified by brown nuclear staining). Scale = 100 µm. Abbreviations: c, capsule; m, medulla.
(TIF)

Figure S2 **Distribution of BrdU-positive cells across the adrenal cortex after different chase periods (preliminary experiment).** Quantitative results of a preliminary experiment to optimise the chase period for identification of label-retaining cells in adult mouse adrenal glands. The percentage of BrdU-positive cells (identified by immunohistochemistry) in each row of a 10 × 10 grid is shown after a 1-week exposure to BrdU followed by chase periods of (A) 4 hours, (B) 5 weeks, (C) 7 weeks, (D) 12 weeks and (E) 15 weeks (1 mouse per chase period). Row 1 is close to the capsule and row 10 is close to the medulla.
(TIF)
