# Supplementary figures and images for: Cell Proliferation, Movement and Differentiation during Maintenance of the Adult Mouse Adrenal Cortex
Source: PLoS One. 2013 Dec 4;8(12):e81865. doi: 10.1371/journal.pone.0081865 (PMC3852665; doi:10.1371/journal.pone.0081865)

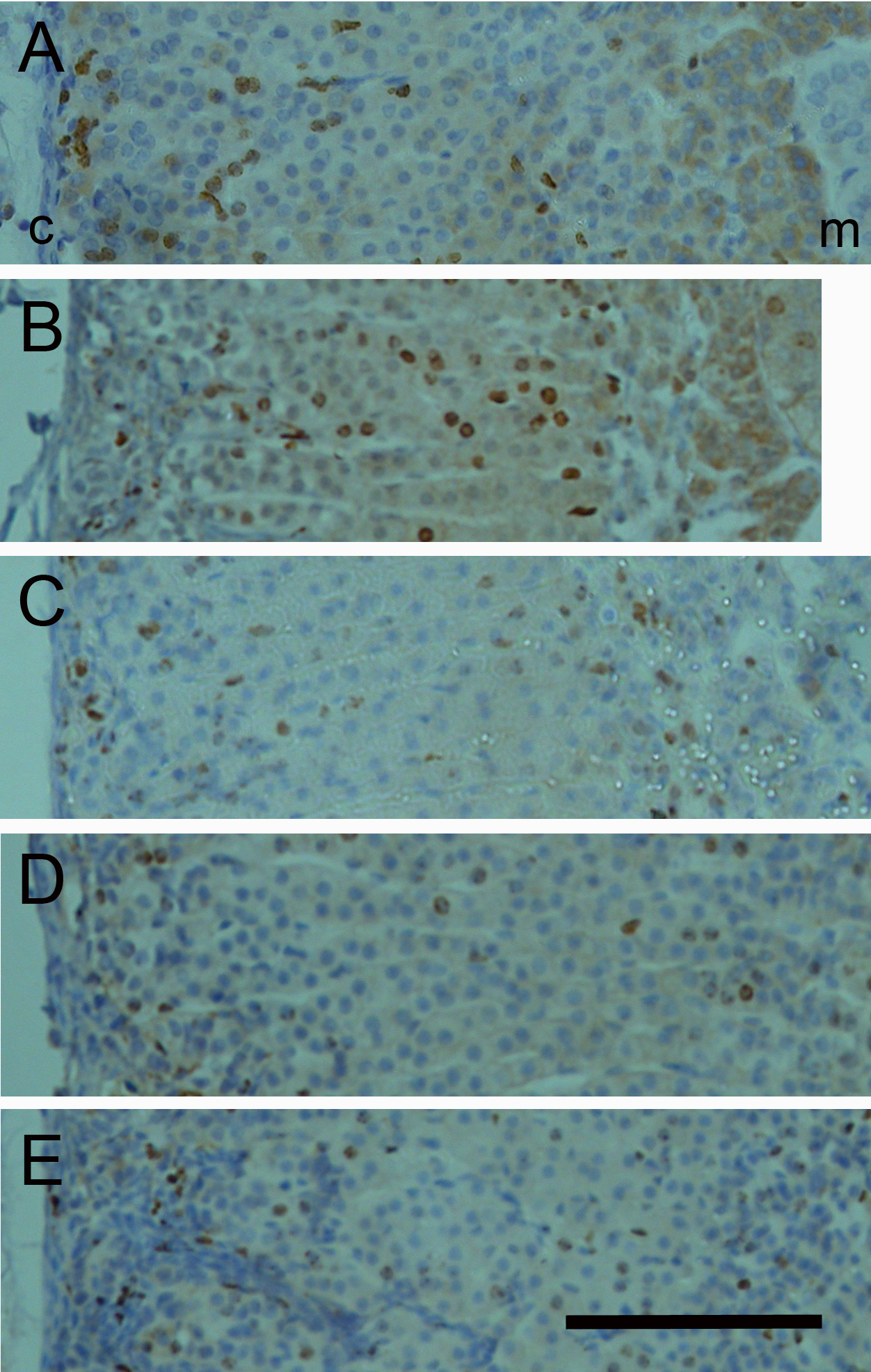

Supplement: Figure S1 — Preliminary BrdU pulse-chase experiment to optimise the chase period for identifying label-retaining cells. (A-E) BrdU immunostaining of adrenal sections from female F1 mice, (A) 4 hours, (B) 5 weeks, (C) 7 weeks, (D) 12 weeks and (E) 15 weeks following a 1-week BrdU infusion, showing distributions of BrdU-labelled cells (identified by brown nuclear staining). Scale = 100 µm. Abbreviations: c, capsule; m, medulla. (TIF) [file pone.0081865.s001.tif]

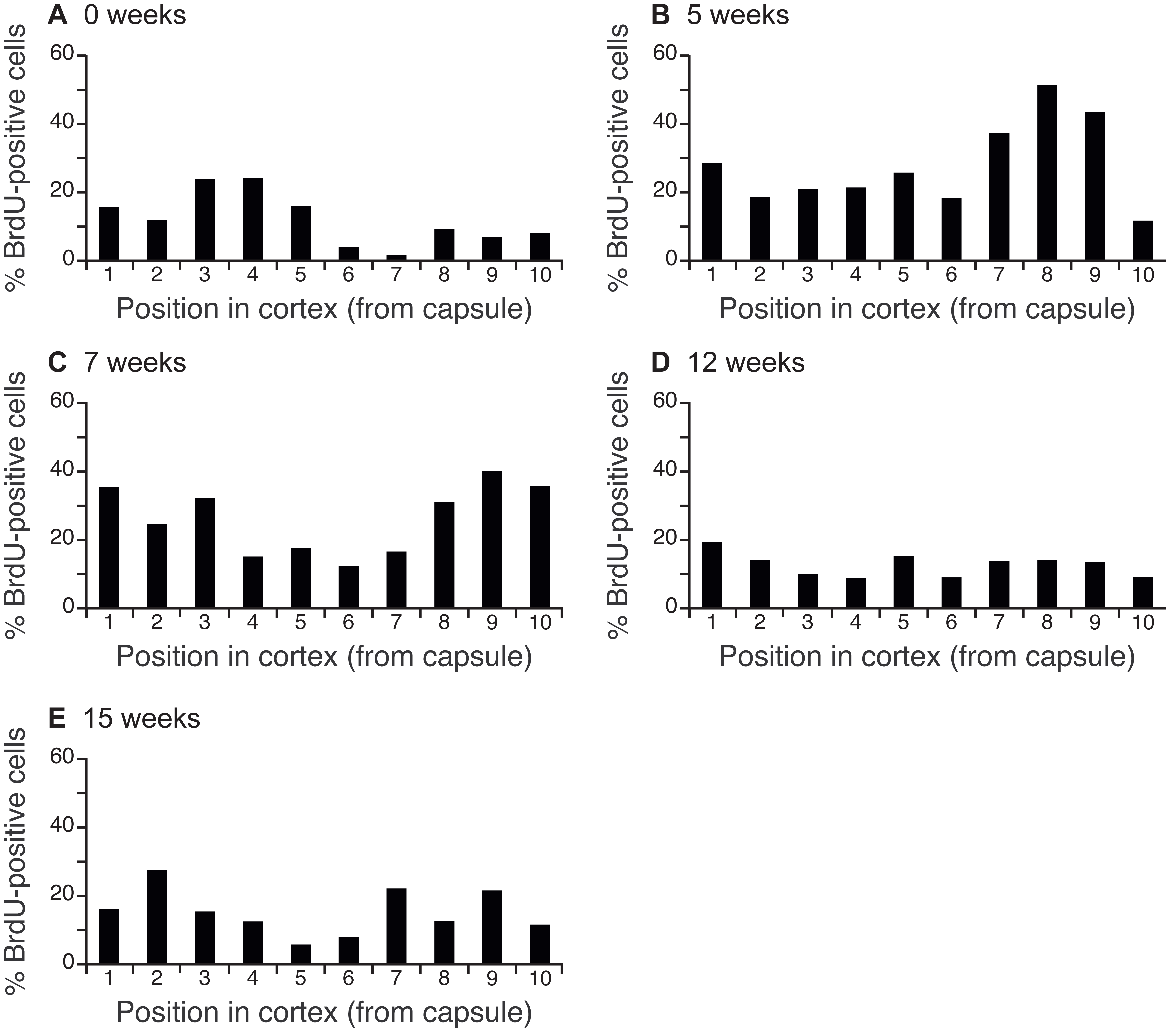

Supplement: Figure S2 — Distribution of BrdU-positive cells across the adrenal cortex after different chase periods (preliminary experiment). Quantitative results of a preliminary experiment to optimise the chase period for identification of label-retaining cells in adult mouse adrenal glands. The percentage of BrdU-positive cells (identified by immunohistochemistry) in each row of a 10 × 10 grid is shown after a 1-week exposure to BrdU followed by chase periods of (A) 4 hours, (B) 5 weeks, (C) 7 weeks, (D) 12 weeks and (E) 15 weeks (1 mouse per chase period). Row 1 is close to the capsule and row 10 is close to the medulla. (TIF) [file pone.0081865.s002.tif]
